# A telephone based assessment of the health situation in the far north region of Cameroon

**DOI:** 10.1186/s13031-020-00327-4

**Published:** 2020-11-30

**Authors:** Etienne Marc Hugues Gignoux, Olivier Tresor Donfack Sontsa, Ayoola Mudasiru, Justin Eyong, Rodrigue Ntone, Modeste Tamakloe Koku, Dalil Mahamat Adji, Alain Etoundi, Yap Boum, Christine Jamet, Jean-Clément Cabrol, Klaudia Porten

**Affiliations:** 1 Epicentre, Paris, France; 2 Médecins Sans Frontières, Geneva, Switzerland; 3 Ministry of Public Health, Yaounde, Cameroon

**Keywords:** Mortality, Telephone, Conflict, Healthcare

## Abstract

**Background:**

In 2017, Field access was considerably limited in the Far North region of Cameroon due to the conflict. *Médecins Sans Frontieres* (MSF) in collaboration with Ministry of health needed to estimate the health situation of the populations living in two of the most affected departments of the region: Logone-et-Chari and Mayo-Sava.

**Methods:**

Access to health care and mortality rates were estimated through cell phone interviews, in 30 villages (clusters) in each department. Local Community Health Workers (CHWs) previously collected all household phone numbers in the selected villages and nineteen were randomly selected from each of them. In order to compare telephone interviews to face-to-face interviews for estimating health care access, and mortality rates, both methods were conducted in parallel in the town of Mora in the mayo Sava department. Access to food was assessed through push messages sent by the three main mobile network operators in Cameroon. Additionally, all identified legal health care facilities in the area were interviewed by phone to estimate attendance and services offered before the conflict and at the date of the survey.

**Results:**

Of a total of 3423 households called 43% were reached. Over 600,000 push messages sent and only 2255 were returned. We called 43 health facilities and reached 34 of them. In The town of Mora, telephone interviews showed a Crude Mortality Rate (CMR) at 0.30 (CI 95%: 0.16–0.43) death per 10,000-person per day and home visits showed a CMR at 0.16 (0.05–0.27), most other indicators showed comparable results except household composition (more Internally Displaced Persons by telephone).

Phone interviews showed a CMR at 0.63 (0.29–0.97) death per 10,000-person per day in Logone-et-Chari, and 0.30 (0.07–0.50) per 10,000-person per day in Mayo-Sava. Among 86 deaths, 13 were attributed to violence (15%), with terrorist attacks being explicitly mentioned for seven deaths. Among 29 health centres, 5 reported being attacked and vandalized; 3 remained temporally closed; Only 4 reported not being affected.

**Conclusion:**

Telephone interviews are feasible in areas with limited access, although special attention should be paid to the initial collection of phone numbers. The use of text messages to collect data was not satisfactory is not recommended for this purpose. Mortality in Logone-et-Chari and Mayo-Sava was under critical humanitarian thresholds although a considerable number of deaths were directly related to the conflict.

## Background

Since March 2014, the far north region of Cameroon has become a conflict zone due to the expansion and influence of Boko Haram. In November 2016 the conflict had already claimed 1500 lives, caused the displacement of 155,000 people and 73,000 refugees [1], in 2017 the humanitarian impact of the conflict worsened with about 4.5 million people without sufficient food. Insecurity limited aid efforts, as Boko Haram carried out 97 suicide and vehicle borne attacks between March and June 2017 according to Nigerian military authorities [2].

In 2016–2017, The Humanitarian Data Exchange platform reported 533 events leadings to 1296 fatalities in the Far North Region [3], as of January 2017 this region hosted over 191,000 IDPs, 23,000 unregistered refugees, and 35,000 returning Cameroonians from Nigeria [4]. This war in the region resulted in huge economical, educational, health and social aftermaths [1, 5, 6] creating an intense humanitarian crisis. The most affected departments were Logone-et-Chari, Mayo-Sava and Mayo-Tsanaga, where the health situation has deteriorated considerably due to the direct consequences of the conflict and reduced access to health care and food [6].

Médecins Sans Frontières (MSF) through its humanitarian mission in the Far North region of Cameroon in collaboration with the Ministry of Public Health (MPH) has been supporting the improvement of the health of populations through: surgical management of patients, maternal and child health, and pediatric and nutritional care for malnourished patients [7]. Because of the prevailing insecurity, MSF has faced considerable restrictions in its access to the field, thus a hindrance to its mission.

MSF needed plausible evidences on health situation in order to better re-orientate its intervention strategies and make strong policies for future actions. Due to the limited access to the population, a classical survey by home visits was evaluated not feasible, therefore we considered the use of mobile phone technologies, which are frequently used for data collection, either by calls or text messages. The method has been widely used in developed countries but is still not common in developing countries especially in Sub-Saharan Africa. Mobile phones stand as a good option to collect data, especially as network coverage and telephone ownership among the populations increases [8]. We felt it was important not only to use this tool to conduct a rapid health assessment in area with restricted access, but also to compare the tool to the traditional face-to-face interviews. In addition to phone interviews, we wanted to test the use of text message questionnaires.

We therefore implemented a health assessment in the populations living in the Logone-et-Chari and Mayo-Sava department using telephone calls, text messages and face-to-face interviews to collect data.

## Methods

### Aim

Our main objective was to estimate mortality rates, health situation, access to healthcare and food insecurity of peoples living in the districts of Waza, Makary and Fotokol (Department of Logone-et-Chari) and in the districts of Tokombere, and Kolofata (Department of Mayo-Sava). We also aimed to compare telephone interviews to face-to-face interviews in urban Mora.

### Design and setting

We used a cross sectional design. This study was conducted in the Far North region of Cameroon, in September 2017. The household telephone survey included 3 districts (Makary, Waza, and Fotokol) in the Logone-et-Chari department; and 2 other districts (Tokombere, and Kolofata) and 1 municipality (urban area of Mora) in the Mayo-Sava department. We used text messages to assess food consumption in the Logone-et-Chari and Mayo-Sava departments. A home visit survey with face-to-face interviews was also conducted in households in the urban area of Mora in order to compare it with the telephone interviews method. A second phase of the survey was done by telephone on health care structures in Makary, Mora, Waza, Fotokol, Tokombere and Kolofata.

The primary outcome of the telephone survey and home visit survey was Crude Mortality Rate (CMR) over a 6 months recall period. We estimated that 30 clusters of 19 households (570 households) in each department and in urban Mora + 4 replacement clusters were needed. The primary outcome of the text message survey was the prevalence of food insecurity, assuming a non-respondent rate of 50% we expected to include a minimum 357 households per department. For the Health care facilities survey we did an exhaustive sampling of all legal health care facilities in the study area.

We did a 2-stage cluster sampling (villages and households) in Waza, Makary, Fotokol, Tokombere and Kolofata. We selected 30 villages (primary sampling units) by department from available administrative lists by Probability Proportional to Size of the populations (PPS) of the villages or neighborhoods (when the village included more than 200 Households). As secondary sampling units, we did a systematic random selection of 19 telephone numbers belonging to household heads from readily available lists that were collected by CHWs in the villages selected. We did a systematic sampling of 1158 telephone numbers of household’s heads in readily available lists from urban Mora that were previously collected by Community Health Workers (CHWs) as described. We used spatial sampling to sample households that will be interviewed by home visit in the urban area of Mora.

For the text message survey, we requested the tree main telephone companies to send push messages to costumers that have been connected 30 days prior to the survey in Logone-et-Chari and Mayo-Sava, informing them about the study. In case of non-response, reminder messages were sent 24 h later. A random sample was drawn from the list of all those who responded to the push message. A web based platform was used to send the questions by text messages to randomly selected participants and for analysis. We included all legal health structures in the study area from lists obtained from health districts heads, and partner NGOs.

### Data collection and analysis

Surveyors underwent a 4 days training (including a 1 day pilot test), which covered the following: survey objectives, ethical issues in surveys, how to conduct survey by telephone and home visit and how to collect data on tablets and smartphones. The household was defined as “a group of people living under the same roof and sharing the same meal at least 3 times a week for about a month regardless of family ties”. For the telephone survey, surveyors called each number 3 times over 2 days before declaring it to be either inexistent, incorrect, or absent. For the home visit survey, surveyors had to revisit the household in the evening in case they missed the occupants or the household head during their previous visit. The household head had the responsibility to respond for the whole on the following: Household composition at the day of interview (age, sexe, residents, displaced, refugees, returnees, or mixed), birth, arrivals and departure during the recall period, morbidity (reported according to their own believe) in the past 15 days and health seeking behaviors, reported deaths and causes of death during the recall period, first dose measles vaccine in children between 1 and 5 years old, Mosquito nets usage in children below 5 years, and the quality of the nets (treated or not and with or without holes), number of meals consumed per day at the time of the interview and last year (only text message survey). For health care facilities, we collected information on the following at the time of the interview and for the period of June 2014 from the heads of the structures: the type of facility (public or private), the different operational services, the number of consultations per day, the number of beds occupied/week, the number of births per week, Human resource, Description of the impact of terrorism on the facility’s activities.

Two questionnaires (one for home visits and household telephone surveys; and another for health care facilities survey) were written and coded on XLS forms and hosted on a Kobo server (Kobotoolbox.org); rules were added when coding the questionnaire to reduce systematic errors and inconsistencies during data collection. The data entered was checked and validated by supervisors every evening before being sent to the server. We exported the final datasets as an excel file and imported them to STATA 13 for cleaning and analysis. We used median (interquartile range) and count (percentage) when necessary to describe the data. Crude-Mortality-Rate (CMR)/10,000 person per day was calculated for the recall period as the ratio of the number of deaths over the total person-time, with 95% Confidence interval (CI) taking into account design effect. The recall period ranged from the 11th of February 2017 (National youth Day in Cameroon) to the date of the interview (from September 9th to 24th, 2017). Household members who had left, those who had arrived, and births during the recall period were considered as having contributed to half of the recall period assuming a constant mortality rate over time; for those who died the exact time under observation was used for calculations. Text messages results were analyzed and presented as bar charts.

## Results

We used CHWs to collect 3679, 3630 and 2666 phones numbers of household heads respectively in Logone-et-Chari, Mayo-Sava, and urban Mora. We systematically selected and attempted to call 999, 1298 and 1158 respectively. Among them, we reached 36.2, 35.9, and 46.2% respectively in Logone-et-Chari, Mayo-Sava, and urban Mora over the phone. Respondent rate was 69.1, 64.8, 69.5 and 87.9% respectively in Logone-et-Chari, Mayo-Sava, urban Mora (telephone interviews) and urban Mora (home visits) (Table 1). We collected information on 2599, 2464, 3378 and 2616 household members present at the time of the interview respectively in Logone-et-Chari, Mayo-Sava, urban Mora (telephone interviews), and urban Mora (household visits).

**Table 1:** Response and participation rate for population survey, Cameroun, 2017

	Telephone			Home visit
	Logone-et-Chari	Mayo-Sava	Urban Mora	Urban Mora
Total numbers collected	3679	3630	2666	397
Total called	999	1298	1158	/
Total reached	362 (36.2%)	466 (35.9%)	535 (46.2%)	397 (100%)
Respondent did not consent^a^	67 (18.5%)	103 (22.1%)	100 (18.7%)	41 (10.3%)
Respondent Under aged^a^	11 (3.0%)	10 (2.1%)	10 (1.9%)	7 (1.8%)
Responded out of survey area^a^	34 (9.4%)	51 (10.9%)	53 (9.9%)	/
Total included^a^	250 (69.1%)	302 (64.8%)	372 (69.5%)	349 (87.9%)

^a^ percentage calculated on the total reached.

### Comparison between telephone survey and home visit in urban Mora

Table 2 present the main results of the comparison. Refusals were almost twice more frequent in phone interview. Age and household size were in similar range however they were statistically different. Phone interviews included many more Internally Displaced persons than did the home visits. During home visits, the responder reported more morbidities during the last 15 days, although he or she attended a health facility (public hospitals, health centre, health post, private health centre) less often than phone responders. Measles vaccine coverage was similar if not equal by both methods, while mosquito nets use were more frequently reported in home visit interviews. Crude Mortality Rate, which is the main objective of this survey, was slightly higher by phone interview but not statistically significantly.

**Table 2:** Results of surveys in urban Mora by home visit and by phone interview, Cameroun 2017

	Home visit	Phone interview	***P*** value
**Household description**			
Refusal	10.3% (*n* = 41)	18.7% (*n* = 100)	< 0.001
Internally Displaced People	28.2% (*n* = 778)	48.5% (*n* = 1711)	< 0.001
Median Age	14 (IQR: 7–27)	13 (IQR: 6–25)	< 0.001
Median Household size	9 (IQR: 6–14)	10 (IQR: 8–14)	< 0.001
**Health**			
Sick during the last 2 weeks	26.9% (*n* = 702)	19.5% (*n* = 654)	< 0.001
Access a health facility during the last 2 weeks	8.7% (*n* = 228)	11.4%(*n* = 385)	< 0.001
received 1st dose Measles Vaccine (1 to 5 Years old)	91% (*n* = 314)	90% (*n* = 438)	0.75
Slept under mosquito net last night (under 5 Years old)	84.5% (*n* = 350)	77% (*n* = 437)	0.003
**Mortality rates (death per 10,000 pers.day)**			
Crude Mortality Rate	0.16 [0.05–0.27]	0.3 [0.16–0.43]	0.15
Under Five Mortality Rate	0 [0–0]	0.33 [0.00–0.73]	NA

### Status, morbidity, access to health care and mortality in the two department

Table 3 present the main results of the telephone surveys in Mayo Sava Department and Logone et Chari. The proportion of household entirely composed of displaced peoples was similar in both departments, however in Logone et Chari, almost a third of household were composed of resident and displaced people (or refugee or returnees), actually in this department only half of the households were only composed of resident. Morbidity and access to health care was similar in both departments. The main morbidities in both locations was malaria (67.9% of cause of sickness in Logone et Chari and 53.9% in Mayo Sava). Measles vaccine coverage was below the level needed to prevent epidemic in Logone et Charri.

**Table 3:** Results of survey Logone et Chari and Mayo Sava Department, Cameroun, 2017

	Logone et Chari	Mayo Sava
**Household**		
N	362	466
Refusal	18.5% (*n* = 67)	22.1% (*n* = 103)
Median Household size	12 (IQR: 9–17)	10 (IQR: 7–14)
Resident	52.0% (*n* = 130)	75.8% (*n* = 229)
IDPs	16.8% (*n* = 42)	13.6% (*n* = 41)
Refugee	1.6% (*n* = 4)	0.3% (*n* = 1)
Returness	1.2% (*n* = 3)	2.0% (*n* = 6)
Mixed (more than one categories of residence in same Houshold)	28.4% (*n* = 71)	8.3% (*n* = 25)
**Individuals**		
N	2813	2577
under 5 years	20.3% (*n* = 571)	19.7% (*n* = 507)
**Health**		
Sick during the last 2 weeks	26.2% (*n* = 651, 95% CI: 22.3–27.9, Deff = 2.8)	27.5% (*n* = 677, 95% CI: 24.3–30.1, Deff = 3.4)
Access a health facility during the last 2 weeks	11.9% (*n* = 308, 95% CI: 9.5–14.7, Deff = 4.4)	13.5% (*n* = 332, 95% CI: 11.3–15.9, Deff = 2.9)
Received 1st dose Measles Vaccine (1 to 5 Years old)	77.8% (*n* = 379, 95% CI: 66.9–85.9, Deff = 6.5)	91.4% (*n* = 382, 95% CI: 88.2–93.8, Deff = 1)
Slept under mosquito net last night (under 5 Years old)	88.8% (*n* = 507, 95% CI: 83.9–92.3, Deff = 2.5)	84.2% (*n* = 427, 95% CI: 76.7–89.7, Deff = 4.1)
**Mortality rates (death per 10,000 pers.day)**		
Crude Mortality Rate	0.63 (95% CI: 0.29–0.97, Deff = 2.8)	0.3 (95% CI: 0.07–0.50, Deff = 2.4)
Under Five Mortality Rate	0.48 (95% CI: 0.00–1.01, Deff = 1.9)	0.55 (95% CI: 0.15–0.87, Deff = 1.1)

Of the 55 deaths, the vast majority (34) of deaths occurred at home and only 11 occurred in either a hospital or a health care center. The deceased sought care in district hospitals (*n* = 16), in integrated health centers (*n* = 4) and 28 of them did not seek for health care.

Of these deaths, 9 were attributed to malaria (8 in Logone-et-Chari against 1 in Mayo-Sava), 3 to respiratory tract infections, 5 to diarrhea, 11 to violence (7 in Logone-et-Chari against 4 in Mayo-Sava), 2 to accidents, 11 to other causes (2 sudden deaths, 2 headache, 1 malnutrition, 1 renal disease, 1 heart attack, 1 stomach ache, 1 diabetes, 1 stroke, and 1 pregnancy complications), and 14 unknown causes. Of the 11 violent deaths, terrorist attacks were specifically mentioned in the comments by 7 household heads.

### Food security from the text message survey

Over 600,000 push messages were sent, of which 2255 responded. The questionnaire was sent to 1836 (900 in Logone-et-Chari and 936 in Mayo-Sava) contacts and 878 responded to the first question, of which 789 completed the questionnaire (358 in Logone-et-Chari and 431 in mayo-Sava) .

**Fig. 1:**
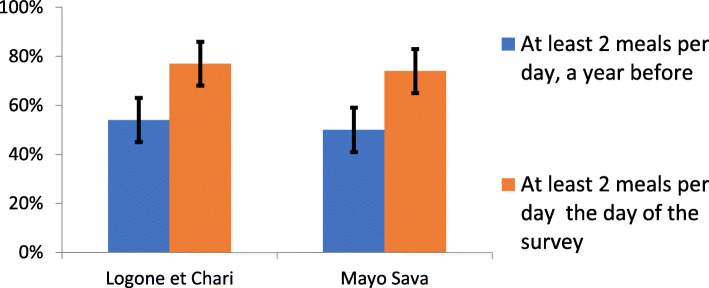
Food access in Logone-et-Chari and Mayo-Sava assessed by text messages, Cameroun, 2017

Food consumption was relatively higher compared to last year (Fig. 1), as 76.2% of the 789 respondents reported having at least 2 meals a day (77.9% in Logone-et-Chari and 74.7% in Mayo-Sava), against 52.6% the previous year.

### Health care facility assessed by telephone interview

From the list of 45 health care structures that we received, we managed to reach 34, of which 29 consented to be interviewed, 2 heads of facilities had been transferred out of the survey area, and 3 refused to participate. Twenty-five of these structures were public (86%) and 4 were private nonprofit. Among the 29 structures that consented to participate, 27 were operational at the time of the interview and 26 in June 2014 (Table 4). Some health structure experienced an increase or a decrease in attendance, which they explained by an arrival of Internally Displaced Peoples or departures. We asked the respondents to describe how the prevailing insecurity situation has impacted on the activities of the structures. Some reported having been attacked or vandalized and temporarily closed, with only a third of them reporting that they had not been affected by the conflict (Table 4).

**Table 4:** Health Facilities In Logonne et Chari and Mayo Sava Department, Cameroun, 2017

	N	%
Identified	45	
Reached	34	75%
Refused to participate	5	14%
Public	25	86%
Atendance decreased	10	34%
Attendance Increased	8	27%
Attacked or vandalized	5	17%
Temporarily closed (up to 1 year)	5	17%
Mentionned fear or insecurity	12	41%
Not affected	9	31%

## Discussions

### Appropriateness and limitations of methods used

#### Telephone versus home visit

We compared telephone and face-to-face interviews for health assessment in urban Mora. The 2 methods showed comparable results for CMR, mosquito nets use, and 1st dose measles vaccine coverage in children; but failed to do so for household composition and morbidity.

The use of information technology including mobile phones, text messages and the internet to collect data is increasingly popular in research, especially in developed countries [9]; and has been applied in many health sectors [10–12]. Early studies showed the comparability of telephone and face-to-face interviews in terms of participation rate and questionnaires completeness [13]; although another study [14] showed that respondent preferred face-to-face interviews as they felt uneasy about discussing sensitive topics over the phone. In another study, both methods were shown to be comparable, although the authors advocated for larger sample sizes when conducting telephone interviews to obtain representative results [15]. Out of 1158 numbers that were called, we reached 46% of which 9.9% fell out of the survey area. Telephone interviews were used in Monrovia to estimate Ebola mortality, where push messages were sent by mobile network operators and those who responded were called; and even though response to push messages was low, the number of participants reached by phone calls was higher [16] than in our study. We used CHWs to collect phone numbers in households and we acknowledge that the very high percentage of unavailability may have been due mistakes during collection; in addition, limited network coverage and malfunctions could have contributed too. Rate of refusals was 18.7% for telephone interviews compared to 10.3% for face-to-face interviews; even though in Monrovia a lower rate of 6.6% was reported [16]. The observed difference between the 2 methods could be explained by the fact that the fact that the populations may be different and thus behaved differently. The insecurity that prevail in the area should not be overlooked, as most the respondent evoked the fact that they don’t talk to strangers or to people they do not know. Despite an almost two-fold higher frequency of refusals in the phone interviews, the proportion of refusals is still low (below 20%) which limits the potential risk of selection bias.

The two interview methods did not to show the same population profiles. There were more households composed of residents by face-to-face interviews. In contrast to the above, a study showed that these methods showed representativeness in the sociodemographic structure of the German population [16]. This discrepancy could be explained by several explanation: misunderstanding of residency over phone, since the survey was conducted in the harvest period residents could have been in farms or at work and thus unavailable or out of phone network, CHWs might have mostly collected telephone contacts of people staying at home the majority of whom may have been IDPs.

CMR was comparable by both methods, although slightly higher by telephone; while no children were reported has having died during the recall period by the face-to-face interviews. Mortality was comparable between a face-to-face survey conducted in Freetown [17] and a telephone survey conducted in Monrovia [16] during the Ebola outbreak; even though contrary to our study, the face-to-face interviews in Freetown showed slightly higher mortality rates. Talking about deaths is a sensitive topic, especially in this region of Cameroon. One might have expected deaths would have been underreported by telephone as a result of uneasiness, but this was not the case. The difference between the 2 methods was not statistically significant, suggesting that telephone interviews are relatively robust to estimate mortality.

During the household telephone survey, we encountered unavailable numbers, numbers that fell out of the study area, and refusals. Although a certain amount of selection bias related to refusals might have been introduced, its impact was limited as frequency of refusals was low. We think that unavailability could be attributed to poor network coverage, insecurity, and social activities (farming). It is also possible that people might have migrated towards other network operators (TIGO from Chad is very popular around Makary and Waza) or may no longer be connected to any. Some numbers fell out of the survey area and could be due to inconsistencies and errors during collection by CHWs.

As an important limitation of our study, was that some lists of numbers collected by CHWs in the selected villages were not complete; households were listed without telephone number. Moreover, in some villages, the numbers of phone contacts collected were far below the estimated number of households, which may be due to low telephone ownership or an incomplete collection process. We recommend that future telephone surveys pay a particular attention to this phase of the study.

#### Text messaging survey

Over half a million push messages were sent to costumers and less than 1% responded. Of the 1836 numbers to which the first question (asking for consent) was sent, only 47.8% (878) responded of which 789 (89%) accepted to participate and completed the questionnaires. We offered an incentive equivalent to 1 $ of free air time for people who accepted to participate to the survey. However, the rate of response to push messages was very low; despite the fact that we had designed radio announcements in preparatory of the survey that were broadcasted in French and in local languages over the national and local radio stations. Regardless of the simplicity of the questionnaire, the rate of participation and completion of the questionnaire was poor. A study of the role of text messaging in research surveys showed low response (7%) and completion (3.6%) rates [18]. Food safety issues can be sensitive and participants may not feel comfortable answering them via text message. However, one study showed that in a cohort that had already agreed to respond to a survey [19], text message or face-to-face responses were similar for health-related questions, suggesting that the low rate of respondents is not related to the nature of the questions but to the method of inclusion. The targeted region is French-speaking, but many local languages are spoken there, and sometimes very locally. The invitation message could only be in written in one language, in this case French, it is possible that the language barrier partially explains the low response rate. Push messages were sent by network operators and as such it was impossible to characterize those who responded, which increases the level of selection bias. The response rate to push messages was very low (< 1%), making the representativeness of the sample questionable.

#### Health facilities telephone survey

List of health care structures were obtained from partner NGOs, and head of health districts. It is possible that not all health care structures were included, making the exhaustibility of the lists questionable. A total of 318 health care structures were reported in 2011 in the Far North region of Cameroon [20], which gives a ratio of 10,950 people per health facility. Applying this ratio on the population covered by our survey we would expect 25 health facilities. We included 45 health care facilities this suggests that we reached most of the health facility in our surveyed area. Health facilities survey was simple to implement and well accepted by responder (14% refusal), it gave useful information directly usable by MSF teams.

### Morbidity, health access, food security and mortality in Mayo Sava and Logone et Chari

The population was very young with a balance in gender. Households in Logone-et-Chari were more composed of IDPs and mixed populations compared to Mayo-Sava where households had more residents. Disease prevalence was 26% with malaria being most prevalent in general and in children less than 5 years. Self-medication was the most common health seeking behavior (45%) and was comparable in both departments. Measles vaccine coverage was 84.1% in general and was lower in Logone-et-Chari than in Mayo-Sava (77.8% vs. 91.4%). Mosquito net usage in children less than 5 was 86.6% and was comparable for both departments. CMR was 0.47 (95% CI: 0.26–0.68) per 10,000 people per day and U5MR was 0.51 (95% CI: 0.15–0.87) per 10,000-person per day; and both were comparable in the 2 departments. The main causes of death were malaria and violence and were more numerous in Logone-et-Chari than in Mayo-Sava.

Disease prevalence was high, and malaria was the main disease in both departments during the survey, which occurred during the rainy season. The high incidence rate of Malaria, especially in children, was also found in other survey and studies in Cameroun and north east Nigeria [21–24]. Despite the high prevalence of malaria, we found a relatively high coverage of mosquito net use (86%) among children under 5 years of age. While a high proportion of the population used self-medication, 48% of the sick population reported to have visited a health care facility. The quality of health care may be questioned as we observed a high rate of attendance relatively to poor human resources and logistics reported in the health facility survey. The Far North region of Cameroon has one of the worst densities of health personnel-to-population [20], the conflict has aggravated the weakness of the health care system, due to the conflict some centers have seen an increases in attendance while others have seen a decrease; others reported to have been vandalized and some have remained closed for some time, only a third of the facilities reported not to have been affected by the conflict (Table 4).

The Far North region of Cameroon and especially the department of Logone-et-Chari is mostly affected by food insecurity (69%) [25]. Surprisingly food consumption was higher than expected and better than last year. This could be linked to the presence of humanitarian NGOs that help the populations through food donations. It was reported that 21% of households in the area were receiving Humanitarian aid [25].

We found mortality rates in children aged below 5 years and in adults below the humanitarian emergency thresholds [26] and most deaths occurred in Logone-et-Chari, compared to Mayo-Sava; this is not surprising since Logone-et-Chari was subject to a more terrorist attacks [27]. A Study done at the same period on nutritional status and retrospective mortality in the northern part of Cameroon reported a CMR of 0.19 and an U5MR of 0.43 in the Far north [28]; which are below our findings and could be explained by the fact that we conducted this study in high risk areas. In general, 16% [9] of the death were attributed to malaria. This observation corroborates the high morbidity of malaria observed. However, violence (20%) was amongst the first causes of death and 7 deaths out of 11 were directly related to terrorist attacks. Most of the violent deaths occurred in Logone-et-Chari (7/11) and could be explained by the high level of terrorist activitiy in the area.

### Other limitations

As with any retrospective survey, the results are subject to recall bias. Death is not easily forgotten, but recall and identification of a disease are prone to memory bias and misclassification. Dates are difficult to remember that could potentially bias the age of the member of household or the death included in the recall period. However, a calendar of events was used by the surveyors to minimize this risk. Numerous studies have shown the lack of reliability of vaccination cards and parental recall to estimate vaccination coverage [29, 30]. Most of the respondents did not understand or speak French, which required the services of local translators, so we recruited surveyors and translators that were experienced. Surveyors were trained and most of them could also speak and understand several local languages (Mandara, Arabic, Hausa, Kanuri, and Foufoulde).

## Conclusions

Telephone interviews are feasible in areas with limited access, although a special attention should be paid to the initial collection of phone numbers. We found discrepancies in population profiles and morbidity between results obtained by phone interview and by home visit; however, mortality which is the main indicator of the severity of humanitarian crisis was comparable for both methods. The response rate to text messages was very low that potentially introduce a strong selection bias, therefore the method as implemented cannot be recommended. Health facilities survey by phone interview proved to be a simple and easy way to collect useful and timely information about health facilities and the difficulties they face.

Malaria was the most frequent cause of mortality after violence. Regardless of the relatively low mortality rates, an important proportion of deaths were caused by violence and most were directly related to terrorist attacks, in a context of limited access to health care. This study shows a clear picture of the situation and challenges faced by health care facilities in Logone-et-Chari and Mayo-Sava. It brings to light the precariousness of the type of services offered, staff, equipment and logistics especially with the massive arrival of refugees, IDPs, and returnees.

## Data Availability

The minimal data set underlying the findings of this study are available on request, in accordance with the legal framework set forth by Médecins Sans Frontières (MSF) data sharing policy (Karunakara U, PLoS Med 2013). MSF is committed to share and disseminate health data from its programs and research in an open, timely, and transparent manner in order to promote health benefits for populations while respecting ethical and legal obligations towards patients, research participants, and their communities. The MSF data sharing policy ensures that data will be available upon request to interested researchers while addressing all security, legal, and ethical concerns. All readers may contact the generic address data.sharing@msf.org or Ms. Aminata Ndiaye (aminata.ndiaye@epicentre.msf.org) to request the data.

## Abbreviations

CHWs: Community health workers
CI: Confidence interval
CMR: Crude mortality rate
MPH: Ministry of public health
MSF: Médecins sans frontieres
PPS: Probability proportional to size of the populations
